# Incorporating frailty and disease severity into treatment decisions for older patients with ANCA-associated vasculitis

**DOI:** 10.1093/rheumatology/keag275

**Published:** 2026-05-25

**Authors:** Mark E McClure, Ayonija Sundararajan, Karl Emil Nelveg-Kristensen, Maya Leibowitz, Matthew L Coates, Dominic McGovern, Michael Chen-Xu, Iftach Sagy, Karol Granak, Tom Quarrell, Shreehari Suresh, Martha Watson, Andreas Kronbichler, Lisa Willcocks, Rona Smith, David R W Jayne, Rachel B Jones

**Affiliations:** Vasculitis and Lupus Clinic, Addenbrooke’s Hospital, Cambridge University Hospitals, Cambridge, UK; Department of Medicine, University of Cambridge, Cambridge, UK; Centre for Public Health, Queen’s University Belfast, Belfast, UK; Vasculitis and Lupus Clinic, Addenbrooke’s Hospital, Cambridge University Hospitals, Cambridge, UK; Department of Nephrology, Rigshospitalet, Copenhagen University Hospital, Copenhagen, Denmark; Department of Medicine, University of Cambridge, Cambridge, UK; Vasculitis and Lupus Clinic, Addenbrooke’s Hospital, Cambridge University Hospitals, Cambridge, UK; Department of Medicine, University of Cambridge, Cambridge, UK; Vasculitis and Lupus Clinic, Addenbrooke’s Hospital, Cambridge University Hospitals, Cambridge, UK; Department of Medicine, University of Cambridge, Cambridge, UK; Vasculitis and Lupus Clinic, Addenbrooke’s Hospital, Cambridge University Hospitals, Cambridge, UK; Department of Medicine, University of Cambridge, Cambridge, UK; Vasculitis and Lupus Clinic, Addenbrooke’s Hospital, Cambridge University Hospitals, Cambridge, UK; Vasculitis and Lupus Clinic, Addenbrooke’s Hospital, Cambridge University Hospitals, Cambridge, UK; Jessenius Faculty of Medicine in Martin, Comenius University in Bratislava, Martin, Slovakia; Department of Medicine, University of Cambridge, Cambridge, UK; Department of Medicine, University of Cambridge, Cambridge, UK; Department of Medicine, University of Cambridge, Cambridge, UK; Vasculitis and Lupus Clinic, Addenbrooke’s Hospital, Cambridge University Hospitals, Cambridge, UK; Department of Medicine, University of Cambridge, Cambridge, UK; Department of Internal Medicine IV, Nephrology and Hypertension, Medical University Innsbruck, Innsbruck, Austria; Department of Health, Medicine and Caring Science, Linköping University, Linköping, Sweden; Vasculitis and Lupus Clinic, Addenbrooke’s Hospital, Cambridge University Hospitals, Cambridge, UK; Vasculitis and Lupus Clinic, Addenbrooke’s Hospital, Cambridge University Hospitals, Cambridge, UK; Department of Medicine, University of Cambridge, Cambridge, UK; Vasculitis and Lupus Clinic, Addenbrooke’s Hospital, Cambridge University Hospitals, Cambridge, UK; Department of Medicine, University of Cambridge, Cambridge, UK; Vasculitis and Lupus Clinic, Addenbrooke’s Hospital, Cambridge University Hospitals, Cambridge, UK; Department of Medicine, University of Cambridge, Cambridge, UK

**Keywords:** ANCA-associated vasculitis, frailty, elderly, rituximab, cyclophosphamide, glucocorticoids, infection, mortality, remission induction, comorbidity

## Abstract

**Objective:**

Older patients with anti-neutrophil cytoplasmic antibody (ANCA)-associated vasculitis (AAV) are underrepresented in clinical trials, and long-term outcome data in this group are limited. We evaluated treatment approaches and outcomes among hospitalized older patients with AAV.

**Methods:**

We retrospectively collected clinical and laboratory data on all hospitalized patients aged 75 years or older who commenced remission induction with cyclophosphamide- or rituximab-based regimens for severe AAV between 2014 and 2021.

**Results:**

Eighty-four patients were included (median age 79, range 75–93), with a median follow-up of 21 months (interquartile range 12–44). Choice of induction regimen was influenced by age and frailty: the rituximab–cyclophosphamide combination was more commonly used in younger patients within the cohort, while low-dose rituximab was favoured for the oldest and most frail, including those requiring residential or nursing home care. The distribution of regimens was as follows: rituximab–cyclophosphamide combination (11.9%), cyclophosphamide (26.2%), standard-dose rituximab (39.6%) and low-dose rituximab (22.3%). Serious infection requiring hospital readmission occurred in 27% of patients within the first year, with rates of 37.5%, 23.8%, 25.8% and 31.6% across the respective treatment groups. One-year mortality was 20% overall (by treatment group: 10%, 23%, 14% and 26%). Increasing age was associated with higher mortality (hazard ratio [HR] 4.15; 95% CI: 1.62, 10.6), but not with serious infection (HR 1.18; 95% CI: 0.47, 2.93).

**Conclusion:**

Considering the enrichment for hospitalized patients with severe disease and advanced age, a mortality rate of 20% at 1 year that is comparable to less severe and younger cohorts suggests tailoring remission induction strategy according to a holistic assessment of frailty and disease activity may partially mitigate the higher risk of infection and mortality with advancing age.

Rheumatology key messages

- Holistic assessment of frailty and disease severity may help guide remission induction for elderly patients with AAV.
- This study provides real-world data using contemporary remission induction strategies on remission, infection and survival in elderly patients with AAV.

## Introduction

Anti-neutrophil cytoplasmic antibody (ANCA)-associated vasculitis (AAV) is a multi-system autoimmune disease for which immunosuppressive therapy with cyclophosphamide or rituximab in combination with glucocorticoids is recommended for severe disease to limit organ damage and improve survival. Although the peak age of onset is 65–75 years [1], a large proportion of patients >75 years of age contribute to the overall incidence but are under-represented in randomized trials that form the basis of treatment recommendations in AAV guidelines [2]. For example, the mean age of participants in CYCLOPS [3], RAVE [4], PEXIVAS [5] and ADVOCATE [6] trials was 53, 57, 63 and 61 years, respectively.

Cyclophosphamide or rituximab-based remission induction regimens are associated with survival benefit for older patients in observational cohort studies [7, 8]. High-dose glucocorticoid regimens contribute to infection and morbidity risks, and recent trials have suggested that reduced dose glucocorticoids can be used in patients with severe disease without compromising efficacy [5], and even further reductions in glucocorticoids may be made in those with less severe disease [9]. Older patients are particularly vulnerable both to the adverse effects of immunosuppression/glucocorticoids and the disease itself, especially around the time of diagnosis when mortality risk is highest [10]. These are important considerations for the physicians treating older patients with AAV who may have high comorbidity burden and frailty.

In our tertiary centre in Cambridge (UK), the immunosuppressive strategy for each patient is overseen by a dedicated vasculitis inpatient service and multidisciplinary team. For older patients, treatment decisions are tailored according to a combined assessment of frailty (functional status, age, comorbidities) and disease severity. Rituximab induction with early steroid minimization is preferred for frailer patients with high perceived infection risk and when attendance for multiple intravenous cyclophosphamide infusions is impractical or too burdensome for patients with poor mobility. The combination of cyclophosphamide and rituximab is reserved for younger patients with severe disease manifestations. In our vasculitis service, patients hospitalized with active AAV tend to be those with more severe disease requiring urgent treatment compared with patients with less active disease managed in an outpatient setting. Typically, hospitalized patients receive three doses of intravenous (i.v.) methylprednisolone (500 mg) followed by a reduced-dose ‘PEXIVAS’ glucocorticoid protocol for remission induction [5] and glucocorticoid dose is minimized for patients at greatest risk of toxicity (infections, diabetes, osteoporosis), aiming for cessation of glucocorticoids by 3–6 months in most patients. Here, we evaluate the induction strategy and outcomes of a cohort of older (≥75 years) patients hospitalized with severe active AAV.

## Methods

### Patient population and data collection

All patients aged ≥75 years who initiated remission induction treatment with cyclophosphamide or rituximab-based regimens, in hospital, for active AAV between 2014 and 2021 at Addenbrooke’s Hospital (Cambridge, UK) were included in the study. ANCA negative patients and those with double positivity for ANCA and anti-glomerular basement membrane antibodies were excluded.

Patients were identified from the vasculitis service database (hospitalized patients from 2014–2021). Clinical and laboratory data were collected retrospectively using electronic patient records. In accordance with the UK National Health Service Research Ethics Committee guidelines, ethical approval was not required because this work comprises retrospective data, and all treatment decisions were made before our evaluation.

### Disease and treatment definitions

Diagnosis of clinical phenotype (granulomatosis with polyangiitis [GPA] *vs* microscopic polyangiitis [MPA]) followed the definitions from the Chapel Hill Consensus Conference, 2012 [11]. Birmingham Vasculitis Activity Score for granulomatosis with polyangiitis (BVAS-GPA), a validated scoring tool, was used to retrospectively quantify disease activity and define involvement of organ systems [12]. There are 34 weighted items split between 10 domains separated by organ system. Minor items score 1 point; major items score 3 points. As there are 19 minor items and 15 major items, the maximum possible score is 64. Remission was defined by the absence of disease activity attributable to active disease (BVAS-GPA = 0). Relapse was defined as the re-occurrence of disease activity attributable to active vasculitis (BVAS-GPA > 0). The definition for serious infection was subsequent further hospitalizations for intravenous or oral antibiotics following hospital discharge after initiation of induction treatment, and followed the Common Terminology Criteria for Adverse Events (CTCAE) v5.0. This definition excluded the use of antibiotics at the time of induction treatment for vasculitis during initial hospitalization either for suspected or proven concurrent infections.

Based on a combined assessment of frailty (functional status, age, comorbidities) and disease activity, patients received one of four induction protocols: (i) a combination of cyclophosphamide (typically 2 × 10 mg/kg i.v. 2 weeks apart adjusted for kidney function as per CYCLOPS trial protocol—more for refractory disease) given alongside rituximab (2 × 1000 mg 2 weeks apart); (ii) cyclophosphamide alone (6–10 × 10 mg/kg i.v. over 3–6 months; adjusted for kidney function as per CYCLOPS trial protocol); (iii) standard dose rituximab (2 × 1000 mg 2 weeks apart); or (iv) low-dose rituximab (either 1 × 1000 mg or 2 × 500 mg 2 weeks apart) [3]. Those who were given methylprednisolone typically received between one and three consecutive days of intravenous pulses of 500 mg/day; however, a minority of patients who received higher cumulative corticosteroid dose, often for resistant disease, were also included. Standard local practice is to co-prescribe gastric and bone protection, as well as co-trimoxazole for *Pneumocystis jirovecii* pneumonia prophylaxis with both rituximab and cyclophosphamide.

Data on the following major co-morbidities was collected: diabetes mellitus, hypertension, cardiovascular disease, stroke, structural lung disease, non-structural lung disease, previous malignancy, cognitive impairment or another comorbidity. In AAV, the assessment of frailty, a widely used concept that describes a state of increased vulnerability due to adverse health outcomes related to ageing, is important to predict recovery from disease as well as treatment side effects. Recognizing that formal assessment of frailty using validated tools such as Rockwood Frailty Scale [13] or PRISMA-7 [14] was not possible in this retrospective cohort study, an individual’s level of social care (prior to admission) was recorded along with comorbidities as surrogate markers of frailty, as these data were readily available from the electronic patient records.

### Statistical analysis

Unless stated otherwise, data are presented as median and interquartile range. Non-parametric data were compared by the Wilcoxon rank sum test. Survival-time analyses were assessed in adjusted Cox proportional hazard models for the assessment of primary outcomes (readmission to hospital with infection and death). Following variables were included in the model: age, sex, BVAS-GPA score, baseline total corticosteroid dose (the total dose of corticosteroid during 7 days prior and including day 0, including both oral prednisolone and intravenous pulses of methylprednisolone), serum immunoglobulin (IgG), serum creatinine, number of comorbidities and level of social care. To improve the power of the models, continuous variables were categorized into binary variables (dichotomized by median value; reference values for each binary variable are shown in Supplementary Figs S1 and S2). Patients entered each model at time of induction treatment and were followed to either their last recorded clinic visit or death. Unless specified in the text, the proportional hazards assumption, linearity of continuous variables and absence of interaction between variables were fulfilled for each model. A two-sided *P*-value of 0.05 or less was considered significant. Analyses and data management were performed in SAS version 9.4 (SAS Institute Inc. Cary, NC, USA).

## Results

Eighty-four patients were identified with a median follow-up of 21 months (interquartile range [IQR] 12–44). The median age was 79 years (range 75–93) with equal sex distribution. Median number of comorbidities in addition to vasculitis was 3 (IQR 2–4). The majority of patients were living independently without need for social care (78.5%). Seventy-nine percent had newly diagnosed disease, with predominantly MPA (66.6%) and myeloperoxidase (MPO)-ANCA (59.5%). Kidney involvement was common (76.2%): median serum creatinine at presentation was 163 µmol/l; 20% required kidney replacement therapy during their initial hospitalization (Table 1).

**Table 1:** Demographics.

Demographic	Value
*n*	84
Age, median (range), years	79 (75–93)
Follow up, median (IQR), months	21 (12–44)
Male sex, *n* (%)	40 (47.6)
Number of co-morbidities, median (IQR)	3 (2–4)
Level of social care, *n* (%)	
Living at home independently	66 (78.5)
Living at home with a care package	12 (14.3)
Care home resident	6 (7.2)
ANCA, *n* (%)	
MPO	50 (59.5)
PR3	34 (40.5)
Clinical Subtype, *n* (%)	
MPA	56 (66.6)
GPA	28 (33.3)
Indication for induction therapy, *n* (%)	
New	67 (79.8)
Relapse	17 (20.2)
BVAS-GPA, median (IQR)	6 (4–8)
Organ involvement	
Kidney, *n* (%)	68 (76.2)
Creatinine at start, median (IQR), µmol/l	163 (91–341)
Required dialysis at presentation, *n* (%)	17 (20.2)
Lung, *n* (%)	50 (59.5)
Pulmonary haemorrhage, *n* (%)	12 (14.2)
Nerve, *n* (%)	18 (21.4)
ENT, *n* (%)	35 (41.6)
Skin, *n* (%)	16 (19.0)
Joints, *n* (%)	27 (32.1)
Eyes, *n* (%)	11 (13.1)
Admitted to ICU, *n* (%)	6 (7.1)
Induction regimen, *n* (%)	
Cyclophosphamide alone	22 (26.2)
Rituximab low-dose (0.5 g ×2)	19 (22.6)
Rituximab normal dose (1 g ×2)	33 (39.3)
Cyclophosphamide (×1–6) + rituximab (1 g ×2)^a^	10 (10.7)
PLEX, *n* (%)	20 (23.8)
i.v. methylprednisolone at induction, *n* (%)	42 (50)
Total corticosteroid dose at baseline, median (IQR), mg^b^	800 (225–2082)
Cumulative prednisolone exposure over first 3 months, median (IQR), mg^c^	971.2 (1567.5–1905)
Maintenance regimen, *n* (%)^d^	
Oral immunosuppression	19 (27.9)
Rituximab	44 (64.7)
No maintenance therapy	5 (7.3)

a In 4/9 patients, rituximab was added to cyclophosphamide (or vice versa) for refractory disease.

b The total dose of corticosteroid during 7 days prior and including day 0, including both oral prednisolone and intravenous pulses of methylprednisolone.

c Calculation only includes those who survived beyond 3 months (*n* = 74).

d Percentage calculation only includes those who survived beyond 6 months (*n* = 68). BVAS-GPA: Birmingham Vasculitis Activity Score for granulomatosis with polyangiitis; ENT: ear nose and throat; GPA: granulomatosis with polyangiitis; ICU: intensive care unit; IQR: interquartile range; MPA: microscopic polyangiitis; MPO: myeloperoxidase; PR3: proteinase 3; PLEX: plasma exchange.

### Choice of induction regimen

**Figure 1:**
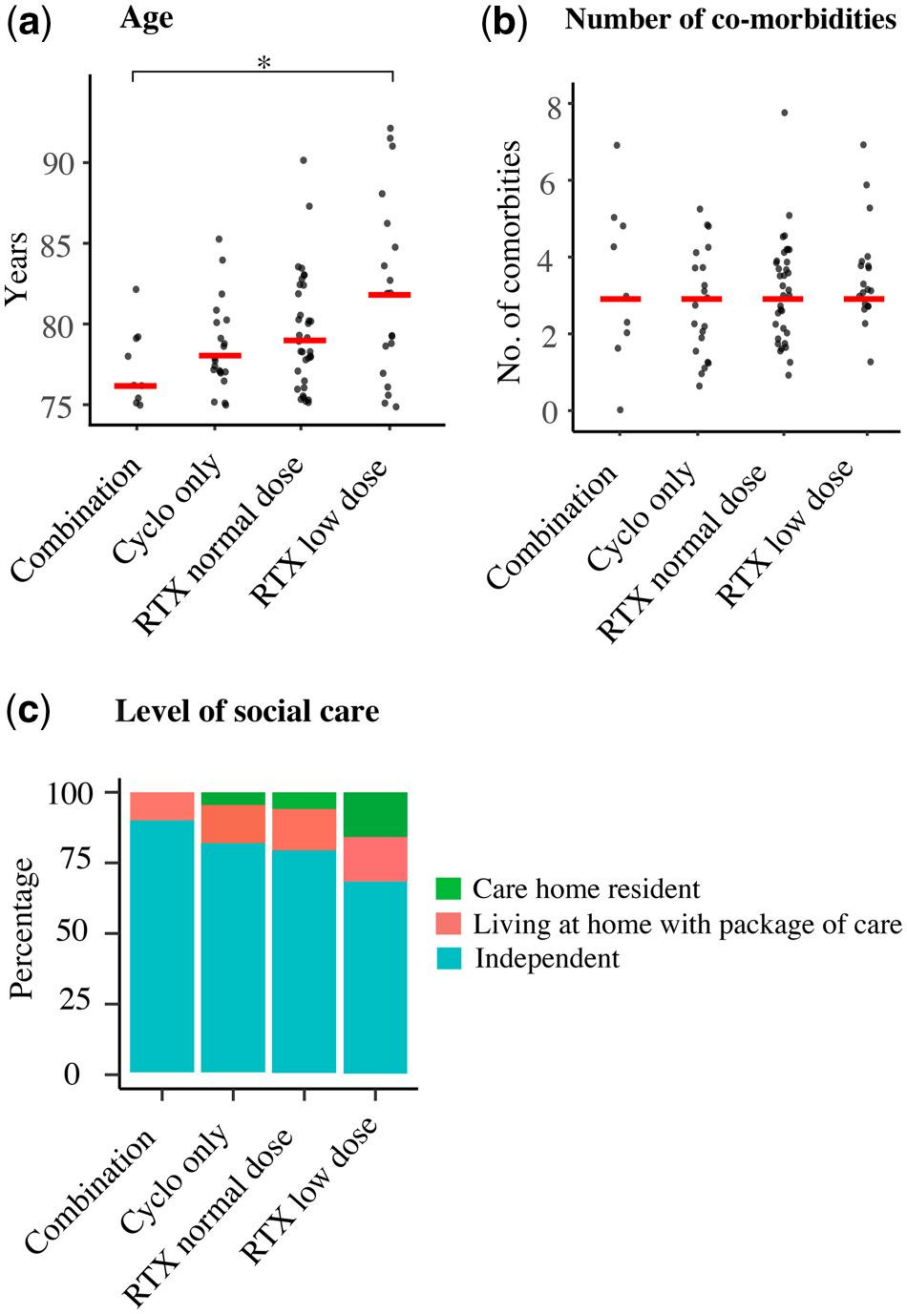
Induction regimen according to age and comorbidities. Dot plots showing the distribution of age (**A**) and number of co-morbidities (**B**), and stacked bar plots divided by level of social care (**C**) for each induction regimen group. Each dot represents one patient; red horizontal lines represent the median values, which are compared between groups using Mann–Whitney *U*-test. Only significant differences are shown: *P < 0.05, **P < 0.01, ***P < 0.001. Cyclo, cyclophosphamide; RTX, rituximab

Induction regimen (rituximab-cyclophosphamide combination [10.7%], cyclophosphamide [26.2%], rituximab standard dose [40%], rituximab low-dose [22.3%]) was age-dependent; the rituximab–cyclophosphamide combination was more frequently administered to relatively younger individuals within the cohort (75–80 years), while low-dose rituximab was more often used in the oldest patients (>85 years). Whilst the absolute number of comorbidities did not appear to influence treatment decisions, patients with frailty defined by the need for residential or nursing home care were more likely to receive rituximab-based regimens (especially low-dose) (Fig. 1).

**Figure 2:**
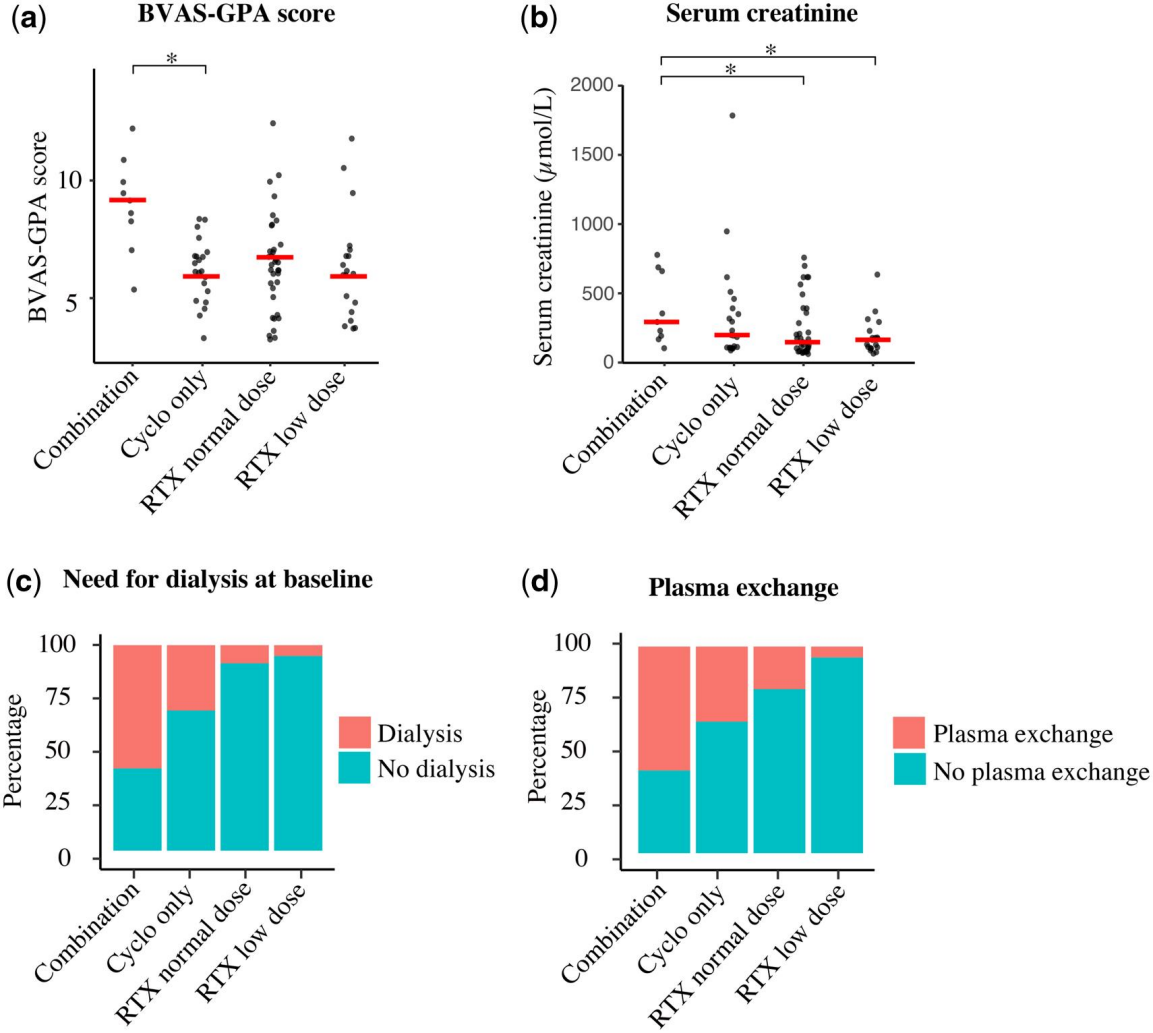
Induction regimen according to disease severity and plasma exchange use. Dot plots showing the distribution of BVAS-GPA score (**a**) and serum creatinine (**b**), and stacked bar plots divided by need for dialysis at baseline (**c**) and adjuvant plasma exchange (**d**) for each induction regimen group. For the dot plots, each dot represents one patient; red horizontal lines represent the median values, which are compared between groups using Mann–Whitney *U*-test. Only significant differences are shown: *P < 0.05, **P < 0.01, ***P < 0.001. BVAS-GPA, Birmingham Vasculitis Activity Score for granulomatosis with polyangiitis; Cyclo, cyclophosphamide; RTX, rituximab

**Figure 3:**
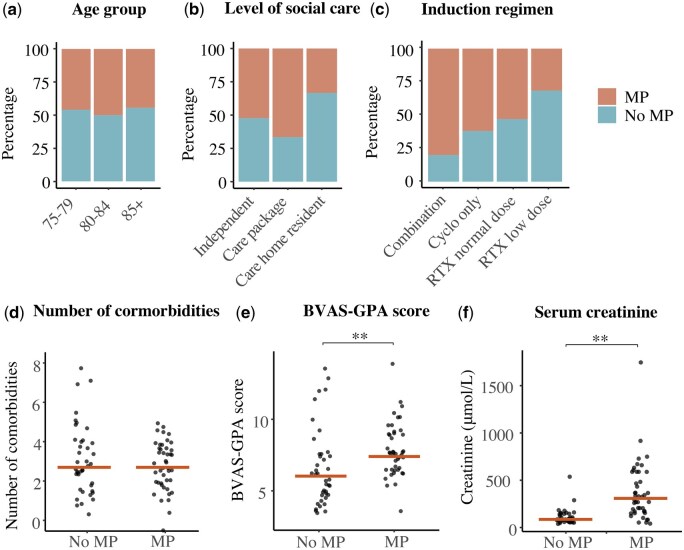
Use of intravenous methylprednisolone. Stacked bar plots divided by age group (**a**), level of social care (**b**) and induction regimen (**c**), and dot plots showing the distribution of number of comorbidities (**d**), BVAS-GPA score (**e**) and serum creatinine (**f**) for those given intravenous methylprednisolone and those who were not. For the dot plots, each dot represents one patient; red horizontal lines represent the median values, which are compared between groups using Mann–Whitney *U*-test. Only significant differences are shown: *P < 0.05, **P < 0.01, ***P < 0.001. BVAS-GPA, Birmingham Vasculitis Activity Score for granulomatosis with polyangiitis; Cyclo, cyclophosphamide; MP, methylprednisolone; RTX, rituximab

Patients who received the combination therapy were more likely to have higher disease activity scores. Either cyclophosphamide alone or in combination with rituximab was preferred in the presence of dialysis dependence/severe kidney involvement (Fig. 2).

i.v. methylprednisolone was used during induction treatment in 50% of patients, and was more commonly prescribed alongside the cyclophosphamide-based regimens (80% and 61% for rituximab–cyclophosphamide combination and cyclophosphamide only, respectively, compared with 52% and 31% for normal-dose rituximab and low-dose rituximab, respectively) in patients with more severe disease activity (BVAS-GPA score 7 *vs* 5, *P* = 0.03) and more severe kidney disease (median creatinine 319 µmol/l *vs* 96 µmol/l, *P* < 0.001). Use of methylprednisolone was not influenced by patient characteristics such as age, level of social care and comorbidities (Fig. 3).

**Figure 4:**
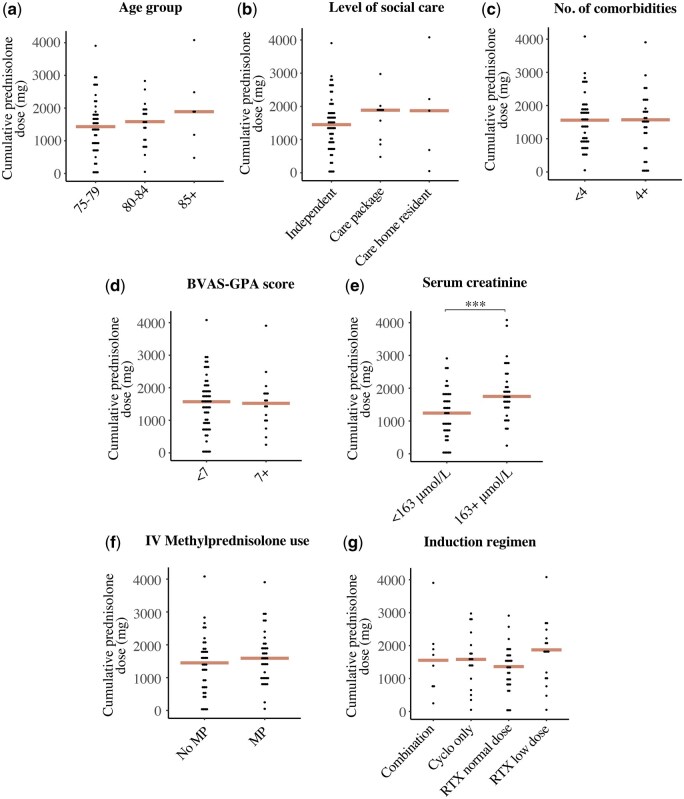
Cumulative oral prednisolone exposure over 3 months. Dot plots showing the distribution of cumulative prednisolone dose over 3 months since induction according to age group (**a**), level of social care (**b**), number of comorbidities (**c**), BVAS-GPA score (**d**), serum creatinine at baseline (**e**), intravenous methylprednisolone use at baseline (**f**), and induction regimen (**g**). Analysis only includes patients who survived beyond 3 months. For the dot plots, each dot represents one patient; red horizontal lines represent the median values, which are compared between groups using Mann–Whitney *U*-test. Only significant differences are shown: *P < 0.05, **P < 0.01, ***P < 0.001. BVAS-GPA, Birmingham Vasculitis Activity Score for granulomatosis with polyangiitis; Cyclo, cyclophosphamide; MP, methylprednisolone; mg, milligrams; RTX, rituximab

The principal determinant of higher cumulative oral prednisolone exposure during the initial 3 months (median 1567.5 mg, IQR 971.2–1905) was the severity of kidney disease rather than patient factors (age, level of social care, comorbidities) or baseline treatments (i.v. methylprednisolone, induction regimen) (Fig. 4).

### Outcomes at 6 months

Thirteen patients (15.5%) died within the first 6 months. The remaining 71 were in complete remission (BVAS-GPA score = 0). Of the 17 (20%) patients who required dialysis at presentation, six (35.3%) remained dialysis-dependent at 6 months, eight (47.1%) were alive and no longer required dialysis, and three (17.6%) had died.

### Infection

Four patients died during their initial admission and were excluded from the infection analysis as the definition for serious infection was readmission to hospital with infection. Out of the remaining 80 patients who survived to hospital discharge, 32 (40%) had at least one serious infection over the course of the follow-up period. Most serious infections occurred early after initiation of induction therapy; 27% of patients had been readmitted with a serious infection within 1 year (37.5%, 23.8%, 25.8% and 31.6% from the combination therapy, cyclophosphamide alone, normal-dose rituximab and low-dose rituximab groups, respectively). None of the pre-specified baseline variables were associated with infection (Supplementary Fig. S1).

### Mortality

Twenty-eight (33.3%) patients died during follow-up, for whom the median time to death was 7 months (IQR 2–18), and included four patients who did not survive to hospital discharge following induction treatment. One-year mortality in the whole cohort was 20% (10%, 23%, 14% and 26% from the combination therapy, cyclophosphamide alone, normal-dose rituximab and low-dose rituximab groups, respectively). Patient age was the only pre-specified baseline variable that was positively associated with increased mortality (hazard ratio 4.15; CI: 1.62, 10.6) (Supplementary Fig. S2).

### Hypogammaglobulinaemia

Moderate hypogammaglobulinaemia (IgG 3.0–5.5 g/l) was observed in 18.8% and 9.4% at 6 and 12 months, respectively. Severe hypogammaglobulinaemia (IgG <3.0 g/l) was observed in 4.3% and 3.7% at 6 and 12 months, respectively. There was no difference in median serum IgG concentration between the induction regimens (Supplementary Fig. S3a); however, moderate/severe hypogammaglobulinaemia (IgG <5.5 g/l) at 6 months was less common in patients who received low-dose rituximab (5%) compared with the normal dose rituximab (21%), cyclophosphamide alone (23%) or combination therapy (28%) (Supplementary Fig. S3b).

## Discussion

The aim of this study was to evaluate the treatment and outcomes of a real-world cohort of older patients with AAV. The choice of induction regimen was influenced by the age, frailty and disease severity (BVAS-GPA and kidney function) of the patient, with a preference for rituximab–cyclophosphamide combination for younger patients with severe (organ threatening) and/or refractory disease, and a low-dose rituximab regimen preferred for older patients who were more likely to be living in a residential or nursing care home.

Whilst interaction between age and treatment regimen highlighted the impact of a patient’s age on the treatment decision, it prevented evaluation of infection risk by treatment regimen in the Cox models. However, when examined individually outside of the Cox models, higher rates of infection were observed in the cyclophosphamide–rituximab combination group compared with the other induction regimen groups, but this did not translate to greater mortality. The decision to use combination therapy was primarily driven by disease severity, which also contributed to the higher infection rates observed in this group. Nonetheless, older age remained an independent and significant risk factor for infection under standardized immunosuppressive regimens, and clinicians deliberately reduced treatment intensity in the oldest and most frail patients. Consequently, patients selected for the most intensive therapy were relatively younger within an overall elderly cohort, a strategy that may have attenuated the infection risk that would otherwise be expected in those with extreme age or frailty. These findings indicate that both disease severity and age/frailty informed treatment selection and infection risk, rather than age alone determining eligibility for intensive therapy.

The 1-year mortality in our study (20%) favourably contrasts with a previously reported elderly AAV cohort [15], which had a 47% mortality rate despite a similar age distribution. Differences in disease severity and management constrain direct comparison—patients in the earlier cohort had more advanced renal impairment (median creatinine 380 µmol/l) and were predominantly treated with oral cyclophosphamide, unlike the more contemporary regimens used in our study. Comparable mortality rates have been observed in a similar aged (but less severe) cohort of AAV patients [16] and a pooled cohort from four EUVAS trials in which the median age was much younger than our study population and frail, older patients were typically excluded [17]. Taken together, this suggests our study has an overall favourable mortality rate compared with prior elderly AAV cohorts considering its enrichment for hospitalized patients with severe disease and advanced age. To put this into context, the 1-year mortality of a 79-year-old (median age of our cohort) in the general UK population is ∼4% [18]; however, for those who require emergency hospitalization, irrespective of diagnosis, 1-year mortality is also ∼20% [19]. In the present study, increasing age was positively associated with mortality but not infection suggesting that selection of induction regimen based on age and frailty may have mitigated the higher risk of infection with advanced age in this cohort. Furthermore, high corticosteroid exposure is associated with increased infection risk, and randomized trials (LoVAS [9], PEXIVAS [5]) have demonstrated that a lower dose of corticosteroid can reduce infections without compromising remission outcomes. Patients in the present study received a lower cumulative prednisolone dose over the initial 3 months (1567.5 mg) compared with both the standard (3150 mg) and reduced (1785 mg) regimens prescribed for a 50–75 kg individual in the PEXIVAS trial (median age 63 years). This reduced steroid exposure may have contributed to the attenuation of infection risk typically observed with advancing age. The availability of avacopan—an oral C5a receptor antagonist that was not available during the study period—now offers the potential to facilitate even earlier corticosteroid withdrawal when used in combination with cyclophosphamide or rituximab-based induction regimens for severe AAV. Although avacopan represents an important advance in steroid-sparing strategies, its impact on infection risk in older patients remains uncertain, as this population was excluded from the ADVOCATE trial and real-world data are currently limited.

Cyclophosphamide and rituximab are both efficacious at inducing remission in patients with AAV [4]. With greater choice of induction agents and glucocorticoid regimens, treatment can be tailored to the individual, considering the risk of organ damage due to disease activity *vs* drug toxicity. Although the infective risks of cyclophosphamide can be reduced by intravenous administration [3] and dose-adjustments for weight, age and kidney function, concern still remains over higher rates of leukopenia, infections and mortality risk in older patients. Consequences of leukopenia may be less severe in young patients as higher rates of leukopenia did not translate to higher rates of infection between cyclophosphamide and rituximab in the RAVE trial [4] where upper age of inclusion was 79 years. Consequently, KDIGO AAV management guidelines [20] recommend rituximab over cyclophosphamide for older patients.

Low-dose rituximab is an attractive therapeutic option for the oldest AAV patients, in whom minimizing immunosuppression and infectious complications is particularly important and less immunosuppression is often required to achieve and maintain remission [21]. In our study, low-dose rituximab was preferred for more dependent patients in whom severe disease was not imminently organ-threatening. Rates of moderate/severe hypogammaglobulinaemia at 6 months after treatment were lowest in the low-dose rituximab group, probably reflecting less immunosuppression. Whilst low-dose rituximab has been successfully used for 6-monthly dosing for remission maintenance in AAV [22], data supporting the use of low-dose rituximab induction in AAV are, to date, limited to case reports and small observational studies, such as a study demonstrating comparable rates of remission at 1 year and no difference in adverse outcomes between low-dose and normal dose rituximab in a Japanese cohort (*n* = 28) [23]. In the current cohort, we do not infer any difference in safety or efficacy between 2 g and 1 g rituximab; rather, the data support reduced dosing as a pragmatic and sufficiently effective option for the oldest and frailest patients.

A further consideration when choosing between rituximab or cyclophosphamide is the presence and extent of kidney involvement. Consistent with other studies [7, 16], we found that kidney involvement was common in this cohort (76%) and often existed with less extra-renal manifestations, possibly resulting in delayed diagnosis and more advanced kidney function impairment at presentation. Post-hoc analysis of patients with kidney involvement from the RAVE trial (*n* = 102) showed no difference in efficacy outcomes between cyclophosphamide and rituximab [24]. However, median serum creatinine concentration of this cohort was 159 µmol/l and patients with a creatinine concentration of above 353 µmol/l were excluded. Thus, for patients with less severe kidney involvement, cyclophosphamide or rituximab is a reasonable option. For patients with more advanced kidney function impairment, the smaller RITUXVAS trial (*n* = 44) provided evidence for cyclophosphamide or cyclophosphamide–rituximab combination therapy [4, 25]. Unlike RAVE, the RITUXVAS trial included dialysis-dependent patients (median entry eGFR 18 ml/min/1.73 m^2^) and showed comparable efficacy between combination of cyclophosphamide (2 × 15 mg/kg i.v.) and rituximab (375 mg/m^2^ × 4) *vs* cyclophosphamide alone (6–10 × 15 mg/kg i.v.) [25]. Although the rates of severe infections in the combination group in RITUXVAS were high (30%), they were comparable to the cyclophosphamide alone group. Infection rates may be improved by combining rituximab with early corticosteroid withdrawal, as demonstrated by a recent observational study in which long-term patient and kidney survival were favourable and infection rates were lower than previously published cohorts treated with purely cyclophosphamide-based regimens [26]. High rates of severe infection in RITUVXAS, where some older patients were included (no upper age for exclusion), led to concern regarding the potential for greater toxicity of rituximab and cyclophosphamide combination in older patients.

The observational design of our study brings both strengths and limitations. Frailty assessment relied on social care status and comorbidity burden as surrogate measures due to the absence of formal frailty scale data. While pragmatic, this approach may underestimate or oversimplify frailty compared with validated tools (e.g. Rockwood, PRISMA-7), as it does not capture all relevant domains of vulnerability. Future research should incorporate standardized frailty instruments prospectively to improve accuracy and clinical relevance. While our cohort reflects the typical demographic of older patients with AAV—characterized by a predominance of MPO-ANCA serotype, MPA phenotype, infrequent ENT involvement, and common kidney disease (with 20% requiring kidney replacement therapy at presentation)—the small sample size from a single institution restricts the statistical power of our models and generalizability to broader populations. This is reflected in the wide confidence intervals, and the interaction between age and induction regimen further complicates conclusions about treatment-specific outcomes. Notably, the cohort selection may introduce bias, as it is likely to have excluded patients with milder presentations who could have been managed in outpatient settings with less toxic oral immunosuppressive agents.

Additionally, the retrospective nature of the cohort introduces important methodological limitations. Data collection depended on chart review, including the calculation of disease activity scores after the fact, which is susceptible to incomplete records and recall bias. This retrospective approach is inherently less robust than prospective assessment and should be considered when interpreting outcomes that rely on accurate disease activity measurement and timing. Finally, cause-of-death data were available for only a minority of patients, predominantly those who died in hospital, as community records or death registry data for patients who died outside hospital were unavailable. Any analysis of infection-related *vs* non-infectious mortality would therefore have been susceptible to ascertainment bias with over-representation of in-hospital deaths. We therefore report overall mortality only and acknowledge the absence of reliable cause-specific mortality data as a further limitation of this study.

## Conclusion

Randomized data on cyclophosphamide and rituximab-based induction regimens for older patients with severe AAV remain limited. Our cohort’s comparable 1-year mortality rate (20%) to younger or less severely affected populations suggests that holistic approaches—tailoring therapy to individual frailty and disease activity—may help mitigate the heightened risks of infection and mortality associated with advancing age.

While future trials specifically designed for older adults are unlikely, it is crucial that studies avoid arbitrary upper age limits in their inclusion criteria. Excluding older patients diminishes the real-world applicability of trial findings and restricts the evidence base needed to guide treatment decisions for older people living with AAV. Randomized trials investigating induction regimens and glucocorticoid use should include patients across all ages, ensuring that study results accurately reflect clinical practice and inform care for everyone affected by AAV.

## Supplementary Material

keag275_Supplementary_Data

## Data Availability

The data underlying this article will be shared on reasonable request to the corresponding author.
