# Supplementary material for: Incorporating frailty and disease severity into treatment decisions for older patients with ANCA-associated vasculitis
Source: Rheumatology (Oxford). 2026 May 25;65(6):keag275. doi: 10.1093/rheumatology/keag275 (PMC13290483; doi:10.1093/rheumatology/keag275)
Supplement: keag275_Supplementary_Data [file keag275_supplementary_data.docx]

**Supplementary Material**

**Supplementary Figure S1. Cox Proportion model for risk of re-hospitalisation with infection**

Multivariable hazard ratios from the Cox proportional hazard model for risk of serious infection after induction treatment for AAV. BVAS GPA, Birmingham Vasculitis Activity Score for granulomatosis with polyangiitis; IgG, immunoglobulin G; HR, hazard ratio; CI, confidence interval. *1, living at home independently; 2, living at home with a care package; 3, care home resident. Reference values were determined by the median value for each variable.

**Supplementary Figure S2. Cox Proportion model for risk of mortality**

Multivariable hazard ratios from the Cox proportional hazard model for risk of death after induction treatment for AAV. BVAS-GPA, Birmingham Vasculitis Activity Score for granulomatosis with polyangiitis; IgG, immunoglobulin G; HR, hazard ratio; CI, confidence interval. *1, living at home independently; 2, living at home with a care package; 3, care home resident. Reference values were determined by the median value for each variable.

**Supplementary Figure S3. Serum IgG at 6 months and rates of hypogammaglobulinaemia**

Dot plots showing the distribution of serum IgG concentrations at 6 months after induction treatment (a), and stacked bar plots divided by rate of moderate/severe hypogammaglobulinaemia (IgG <5.5 g/L) 6 months after induction treatment (b) for each induction regimen group. For the dot plots, each dot represents one patient; red horizontal lines represent the median values, which are compared between groups using Mann Whitney U test. Only significant differences are shown: *<0.05, **<0.01, ***<0.001. Cyclo, cyclophosphamide; IgG, immunoglobulin G; RTX, rituximab.
